# Up-regulation of Synaptotagmin IV within amyloid plaque-associated dystrophic neurons in Tg2576 mouse model of Alzheimer’s disease

**DOI:** 10.3325/cmj.2013.54.419

**Published:** 2013-10

**Authors:** Larisa Tratnjek, Marko Živin, Gordana Glavan

**Affiliations:** 1 Laboratory for Brain Research, Institute of Pathophysiology, Medical Faculty University of Ljubljana, Ljubljana, Slovenia; 2 Department of Biology, Biotehnical faculty, University of Ljubljana, Ljubljana, Slovenia; Tratenjek et al: Up-regulation of Synaptotagmin IV within amyloid plaque-associated dystrophic neurons in Tg2576 mouse model of Alzheimer’s disease

## Abstract

**Aim:**

To investigate the involvement of the vesicular membrane trafficking regulator Synaptotagmin IV (Syt IV) in Alzheimer’s disease pathogenesis and to define the cell types containing increased levels of Syt IV in the β-amyloid plaque vicinity.

**Methods:**

Syt IV protein levels in wild type (WT) and Tg2576 mice cortex were determined by Western blot analysis and immunohistochemistry. Co-localization studies using double immunofluorescence staining for Syt IV and markers for astrocytes (glial fibrillary acidic protein), microglia (major histocompatibility complex class II), neurons (neuronal specific nuclear protein), and neurites (neurofilaments) were performed in WT and Tg2576 mouse cerebral cortex.

**Results:**

Western blot analysis showed higher Syt IV levels in Tg2576 mice cortex than in WT cortex. Syt IV was found only in neurons. In plaque vicinity, Syt IV was up-regulated in dystrophic neurons. The Syt IV signal was not up-regulated in the neurons of Tg2576 mice cortex without plaques (resembling the pre-symptomatic conditions).

**Conclusions:**

Syt IV up-regulation within dystrophic neurons probably reflects disrupted vesicular transport or/and impaired protein degradation occurring in Alzheimer’s disease and is probably a consequence but not the cause of neuronal degeneration. Hence, Syt IV up-regulation and/or its accumulation in dystrophic neurons may have adverse effects on the survival of the affected neuron.

The main pathological hallmarks of Alzheimer’s disease (AD) are the formation of amyloid plaques, neurofibrillary tangles, dystrophic neurites, and sometimes activation of glial cells in the brain (1,2). In the vicinity of amyloid plaques, neurons undergo dramatic neuropathological changes including metabolic disturbances such as altered energy metabolism, dysfunction of vesicular trafficking, neurite breakage, and disruption of neuronal connections (3-8).

Synaptotagmin IV (Syt IV) is a protein involved in the regulation of membrane trafficking in neurons and astrocytes (9,10). In hippocampal neurons, it regulates brain-derived neurotrophic factor release (11) and is involved in hippocampus-dependent memory and learning (12,13). In astrocytes, it is implicated in glutamate release (10). Recent data show that Syt IV plays an important role in neurodegenerative processes (14). Syt IV expression could be induced by seizures, drugs, and brain injury. Its changes have been shown in several animal models of neurodegeneration (Parkinson’s disease, brain ischemia, AD) (14-25). However, the exact role of Syt IV in neurodegeneration is unknown.

Our previous study showed that the expression of Syt IV mRNA and its protein in the hippocampus and cortex of Tg2576 mouse model for AD was increased in the tissue surrounding β-amyloid plaques (14). It is not clear whether Syt IV is expressed in astrocytes (10,26,27) or/and in neurons (28,29), ie, whether it regulates the release of pro- or anti-inflammatory cytokines from β-amyloid associated astrocytes or is involved in neuronal vesicular pathogenesis (5,30). Therefore, the present study aimed to determine the type of cells in which Syt IV up-regulation occurs.

## Methods

### Transgenic animals and tissue preparation

Tg2576 mice, the AD model (31,32), express the human amyloid precursor protein (APP) gene with the Swedish familial 670/671 NL double mutation under transcriptional control of the hamster prion promoter. Tg2576 mice brains together with corresponding wild type (WT) littermate controls of the same genetic background (C57Bl/SJL) were kindly provided by Dr Reinhard Schliebs, Experimental Centre of the Medical Faculty, University of Leipzig, Germany, where breeding was performed in 2011 (30). The founder mice originate from Dr Karen Hsiao, Ashe laboratory (University of Minnesota, USA). The animal experiments were approved by the Independent Ethical Committee of the Regierungsprasidium Leipzig. Animals were handled according to the NIH Guide for the Care and Use of Laboratory Animals.

Immunohistochemical and immunofluorescent staining was carried out on a free floating section of transcardially perfused brains of four Tg2576 mice (19 to 29 months old) and four non-transgenic age-matched mice. Mice were perfused transcardially with cold saline under deep anesthesia, followed by cold 4% phosphate buffered formaldehyde (pH 7.2-7.4). Dissected brains were postfixed by immersion in 20% sucrose in 4% formaldehyde at 4°C and cryoprotected in 20% sucrose in sodium phosphate buffer at 4°C for 48 hours. Coronal brain sections throughout the cortex and hippocampus (between −0.94 mm to −4.04 mm from the bregma) were cut at 20 µm from frozen brain using a freezing-state microtome. Processed free-floating brain slices were stored at -20°C in a cryoprotectant solution.

Western blot analyses were performed on frozen brain slices from four Tg2576 (19 to 29 months old) and four age matched WT mice brains. The brains were rapidly removed and quickly frozen on dry ice. Coronal sections throughout the cortex and hippocampus (between −0.94 mm to −4.04 mm from the bregma) were cut in cryostat into 30-μm sections and then stored at -20°C.

### Syt IV immunohistochemistry and quantitative analysis

Immunohistochemistry was performed as described previously (25). Briefly, coronal brain sections were incubated in sodium citrate solution (pH = 8.5; 30 minutes, 80°C) for antigen retrieval and then in blocking buffer containing 4% normal serum, 1% BSA, and 0.1% Triton X-100 in potassium phosphate buffer (KPBS) for 1 hour at room temperature. They were incubated with rabbit polyclonal primary antibody against Syt IV (1:100, Immuno-Biological Laboratories, Gunma, Japan) overnight at 4°C and then by biotinylated anti-rabbit secondary antibodies (1:500, Vector Laboratories, Burlingame, CA, USA) for 1 hour at room temperature. Avidin-biotin-peroxidase complex (ABC elite standard kit, Vector Laboratories) was added. Staining was visualized with 3,3′-diamino-benzidine (DAB, Aldrich Chemicals, Milwaukee, WI, USA). All sections were immunolabeled simultaneously to ensure the same conditions such as using identical DAB staining incubation times. Sections were then mounted, dehydrated, and coverslipped with DPX mounting medium (BDH Laboratory supplies, Poole, UK). Some sections were directly mounted in Vectashield Mounting Medium containing DAPI (Vector Laboratories). Slices were examined and imaged with Olympus microscope (Olympus IX81, Olympus Optical, Tokyo, Japan) with an attached digital camera (Olympus DP71) using the same system settings for all samples. Omission of the primary antibodies served as negative control.

Syt IV immunohistochemical staining was quantified in Tg2675 (n = 4) and WT mice (n = 4) cortices in three sections per animal. Syt IV immunosignal intensities were determined by measuring immunosignal relative optical density (ROD) around 10 systematically randomly chosen amyloid plaques per section with MCID, M4 image analyzer (Imaging Research Inc., St. Catharines, ON, Canada). For each plaque, three 300 μm^2^ areas were systematically randomly sampled within Syt IV-rich corona around each plaque and three 300 μm^2^ areas in the plaque periphery (within a radius of 20 μm from the edge of Syt IV-rich corona). For every analyzed plaque, immunosignal intensities were also measured in three 300 μm^2^ areas in approximately the same position in cortical plaque-free areas (interplaque tissue) on opposite brain slice hemisphere. Similar procedures were applied to WT mice. Altogether 120 areas of plaques, plaque periphery, interplaque, and WT cortical regions were analyzed. A two-tailed t-test and one-way ANOVA, following Bonferroni’s multiple comparison test was used for statistical analysis (Prism; GraphPad Software, San Diego, CA, USA).

### Western blot

Tissue lysates were prepared by homogenizing cortex and hippocampus from two 30-μm frozen brain slices of WT and Tg2576 mice brains in CelLytic-M Cell Lysis reagent (Sigma, St. Louis, MO, USA). Total protein concentrations were determined with Bio-Rad protein assay quantification kit (Bio-Rad Laboratories, Hercules, CA, USA). Proteins were separated on 10% NuPAGE Bis-Tris Mini Gels (Novex by Life technologies, Carlsbad, CA, USA) and electrophoretically transferred to nitrocellulose membrane (Invitrogen, Carlsbad, CA, USA). Immunodetection of Syt IV was performed employing the WesternBreeze Chemiluminiscent immunodetection system (Invitrogen) and anti-Syt IV antibody (Immuno-Biological Laboratories, 1:100 dilution) according to the manufacturer instructions. After the incubation of membrane with chemiluminiscent substrate, the signal was visualized by exposure of blots to CP-BU x-ray film (Agfa HealthCare NV, Belgium). The Western blot was stripped (0.1 M 2-mercaptoethanol, 2% sodium dodecyl sulfate, 62.5 mM Tris-HCl, all from Sigma) and re-probed again with rabbit polyclonal anti-actin antibody (Sigma, 1:1000 diluted). The relative optical density of the bands was measured using MCID, M4 image analyzer. The densitometric values of the bands representing Syt IV immunoreactivity were normalized to the values of the corresponding actin bends. Western blot analyses were performed on the data from three replicate experiments. Data were analyzed using Graph Pad Prism software. A two-tailed t-test was used to determine statistical significance.

### Double immunofluorescence labeling and quantitative analysis of cellular localization of Syt IV

Sections were incubated with Syt IV antibody (1:100) and one of the following antibodies: mouse monoclonal antibody recognizing astrocytes (GFAP – Glial fibrillary acid protein, 1:2000, Millipore, Bilerica, MA, USA), microglial cells (0X-6 – major histocompatibility complex class II, 1:200, Abcam, Cambrige, UK), neurons (NeuN – neuronal nuclear specific protein, 1:200, Millipore), or neurites (neurofilaments-L (DA2), 1:200, Cell Signaling technology, Beverely, MA, USA). Signals were detected using goat anti rabbit AlexaFluor 488 and goat anti mouse AlexaFluor 555 secondary antibodies (1:200, Invitrogen-Molecular Probes, Eugene, OR, USA). After incubation, slices were immersed into 0.1% Sudan Black B (Sigma-Aldrich, St Louis, MO, USA) in 70% vol/vol ethanol for 5 minutes to suppress lipofuscein background autofluorescence. Sections were then rinsed with KPBS, mounted, and coverslipped using Prolong Gold antifade reagent with DAPI (Invitrogen) for DNA labeling. To confirm staining specificity, primary antibodies were omitted. To examine the possible cross-reactivity between antibodies or bleedthrough between wavelength channels single immunofluorescence labeling was performed.

The co-localization of Syt IV immunofluorescent signal with markers for microglial cells, astrocytes, neurons, and neurofilaments in WT and Tg2576 mice cortex was examined with a fluorescence microscope AxioImager.Z1 (Carl Zeiss MicroImaging GmbH, Heidelberg, Germany) with an ApoTome attachment for optical sectioning in coronal brain sections. The images were captured in a Z-series with an interslice gap of 0.240 using a 60 × 1.4 numerical aperture (NA) oil objective. Double labeled coronal WT brain sections were randomly optically sectioned throughout the cortex; altogether 20 images (14.435 μm^2^ scan field per image) were analyzed for each labeling. In Tg2576 mice cortex, the images were obtained only for representative plaques that could be clearly identified (60 β-amyloid plaques were analyzed for each double labeling) and for interplaque regions (altogether 20 images were analyzed for each labeling).

The total number of cells in each 14 435 μm^2^ cortical field was determined by counting DAPI labeled nuclei. Afterward, the number of cells positive for one of cell-specific markers – GFAP, OX-6, or NeuN was determined in the same cortical fields. Next, we manually counted the double-immunoreactive Syt IV/GFAP, Syt IV/OX-6, and Syt IV/NeuN cells. All procedures were applied to plaque areas within Syt IV-rich corona in interplaque region and in WT cortex. Two-way ANOVA, following Tukey multiple comparison test, was used for statistical analysis (Prism; GraphPad Software).

### Thioflavin S staining

Fibrillar Aβ was visualized by staining with Thioflavin S (Sigma-Aldrich). After Syt IV immunohistochemistry, sections were incubated with a solution of 0.015% Thioflavin S in 50% vol/vol ethanol solution for 10 minutes. Sections were then rinsed with 50% ethanol and incubated with Sudan Black B as described above, rinsed with KPBS, mounted, and coversliped with Prolong Gold antifade reagent with DAPI. Altogether 60 Thioflavin S-positive amyloid plaques were analyzed for Syt IV immunoreactivity.

## Results

### Syt IV is present in the wild type mouse cortex only in the neurons

**Figure 1:**
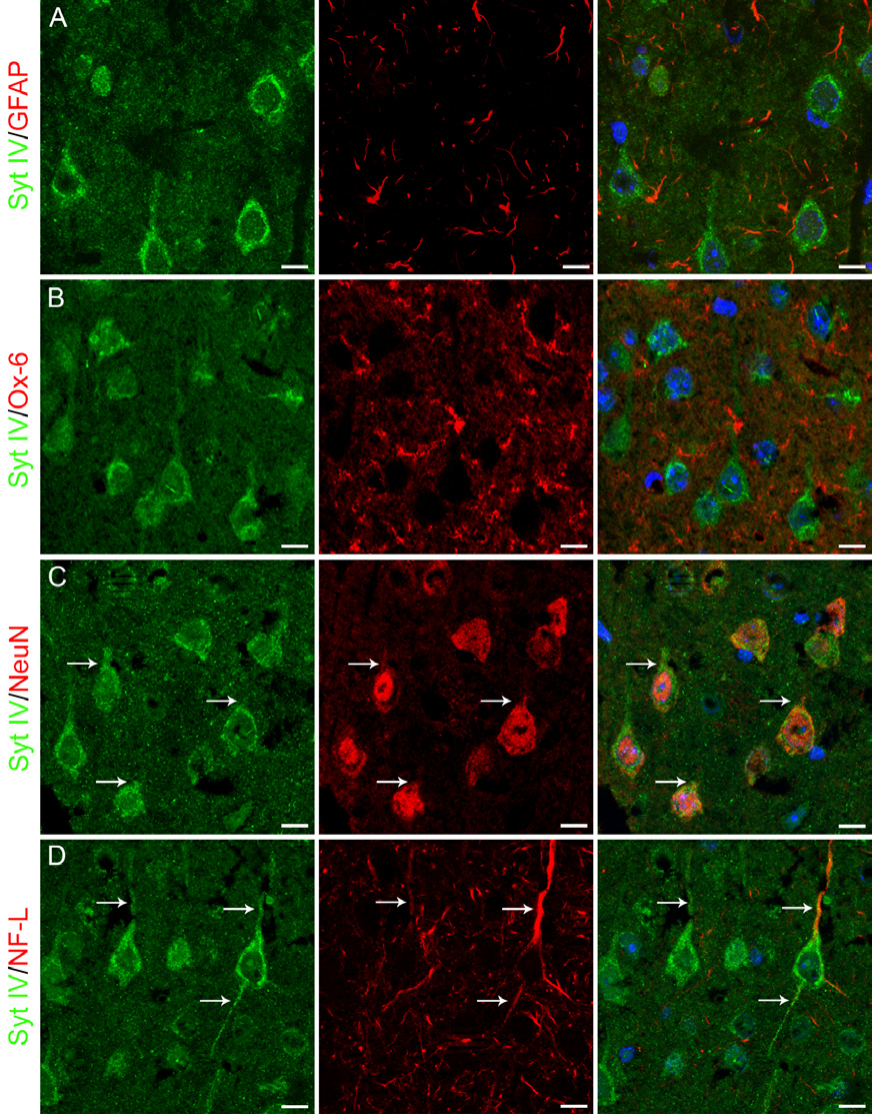
Synaptotagmin IV (Syt IV) was present in neuronal cells in wild type (WT) mice cortex. Sections of WT mice cortex were immunoprobed for Syt IV and markers for astrocytes (GFAP, **A**), microglial cells (Ox-6, **B**), neurons (NeuN, **C**) and neurites (NF-L, **D**). No astrocytes (**A**) or microglial cells (**B**) were positive for Syt IV. All NeuN-positive neuronal cells were Syt IV-immunoreactive (**C**). Syt IV had a cytosolic subcellular distribution and was present along neuronal processes (arrows, **C**). Syt IV co-localized with neurofilaments along axons and dendrites (arrows, **D**). Scale bars, 10 μm.

Double immunofluorescence labeling of Syt IV and markers for astrocytes (Figure 1A) and microglial cells in WT mice (Figure 1B) showed that glial cells were not Syt IV-immunoreactive. On the contrary, double immunofluorescence labeling of Syt IV and markers for neurons revealed strong Syt IV labeling in cortical neurons (Figure 1C). Syt IV had cytosolic localization in neurons and appeared along neuronal processes (Figure 1C) in vesicle-like structures. Thus, labeling with the antibody against neurofilaments showed co-localization of Syt IV with neurofilaments along axons and dendrites (Figure 1D).

### Syt IV is up-regulated around the β-amyloid plaques

**Figure 2:**
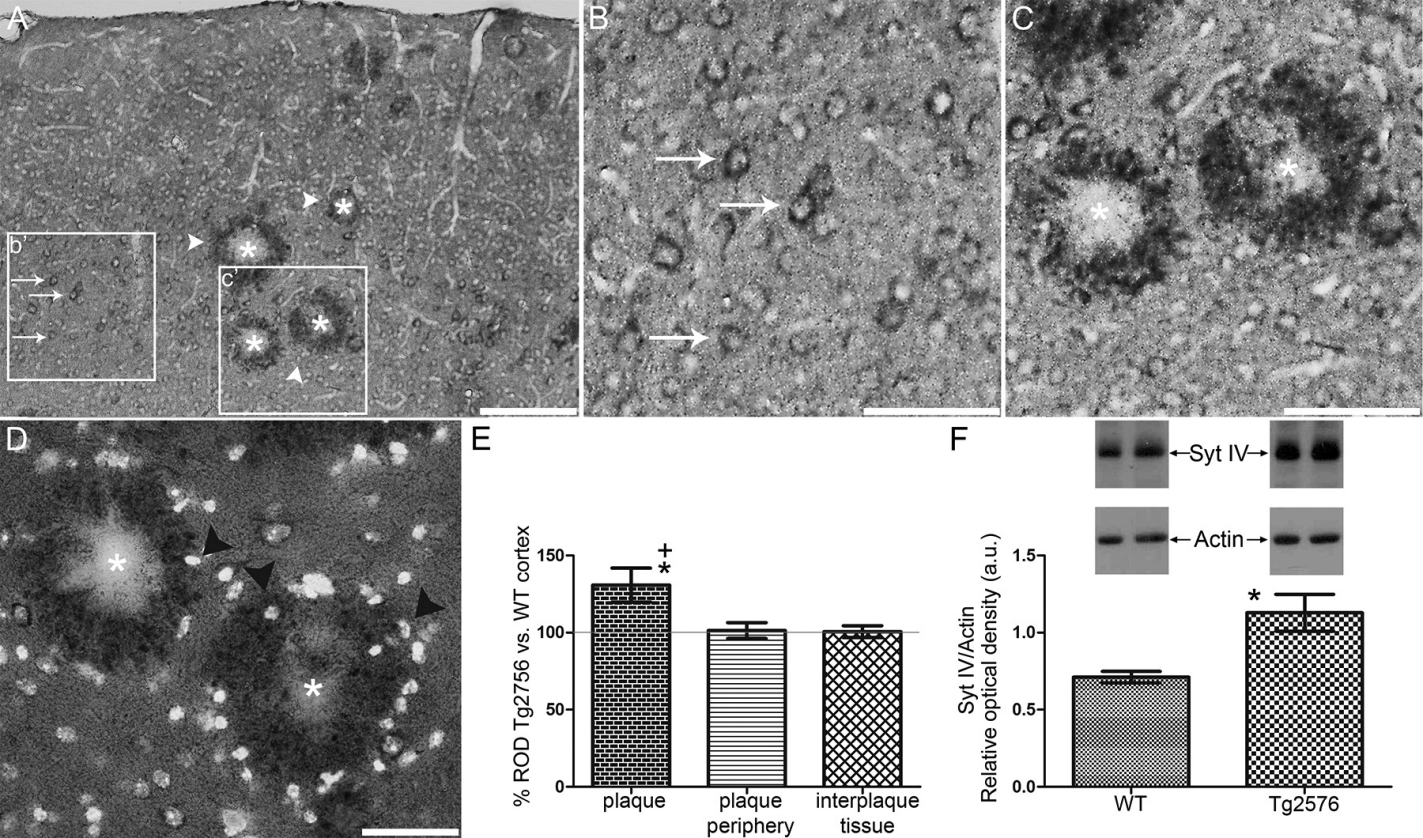
Synaptotagmin IV (Syt IV) was up-regulated in Tg2576 mice brain. Immunohistochemical labeling of Syt IV in cortex of Tg2576 mice revealed cortical neurons (arrows, **A**) and strong up-regulation of Syt IV-β-amyloid plaque-associated immunoreactivity (arrowheads, **A**). A high-magnification view of the boxed area b’ in plaque free areas of transgenic mice cortex in image A is shown in image B. Syt IV-immunoreactive cells in plaque free areas are probably neurons (arrows, **B**). A high-magnification view of the boxed area c’ in image A is shown in image C. Syt IV immunoreactivity was in close proximity of amyloid plaques (asterisks, **A**, **C-D**) and formed a corona of irregular and spherical structures containing up-regulated Syt IV. A merged image of Syt IV labeling and nuclei counterstaining with DAPI (**D**) shows some nuclei present within Syt IV-immunoreactive corona (arrowheads, **D**, but the majority of Syt IV-immunoreactive signal was located between amyloid plaques and cells. Scale bars, 100 μm (**A**), 50 μm (**B-D**). Quantitative Syt IV immunohistochemistry analysis showed increased Syt IV levels around amyloid plaques (**E**), while Syt IV levels were unaltered in plaque periphery (20 μm wide tissue ring around Syt IV-enriched corona) or in interplaque regions (**E**) as compared to corresponding WT cortical areas. The graph shows average relative optical density (ROD) of Syt IV immunohistochemical signal around plaques, in plaque periphery, and in interplaque regions in the cortex of Tg2576 mice (n = 4) expressed in percentages of ROD of Syt IV immunosignal in the corresponding WT cortical areas (n = 4). *Significantly higher compared to Syt IV immunohistochemical signal ROD in the corresponding WT cortical areas (unpaired two-tailed t-test, *P* < 0.0001). ^+^Significantly higher compared to Syt IV immunosignal ROD in plaque periphery and in interplaque tissue (one way ANOVA followed by Bonfferoni’s multiple comparison test, *P* < 0.001). Quantification of Syt IV protein levels in control (n = 4) and transgenic mice (n = 4) cortex and hippocampus by Western blot showed increased Syt IV level in transgenic mice brain compared to WT mice (**F**). Representative blots for Syt IV protein in WT and transgenic animals (**F**). Actin was used as a loading control. Graph (**F**) shows mean relative optical density of Syt IV bends normalized to actin and expressed in arbitrary units (a.u.) (*P = 0.011, unpaired two-tailed t-test). (**E-F**) Error bars represent the standard deviation of the mean.

Immunohistochemical labeling of Syt IV in Tg2576 cortex revealed Syt IV- immunoreactive cortical cells (Figure 2A-B). In addition, strong up-regulation of Syt IV around the β-amyloid plaques (Figure 2A, 2C-D) was observed. The pattern of immunolabeling in WT cortex was similar to the pattern observed in the interplaque area of transgenic mice cortex (Figure 2B). The up-regulated Syt IV immunohistochemical signal was located mostly between amyloid plaques and cell nuclei (Figure 2C-D) forming a corona around plaques. However, there were cell nuclei present within the limits of Syt IV-enriched corona (Figure 2D).

In Tg2576 mice, quantitative Syt IV immunohistochemistry analysis showed a significant increase in the signal around amyloid plaques as compared with the signal in the WT mice cortex (by 30.8 ± 10.96%, unpaired two-tailed t-test, *P* < 0.001, Figure 2E). However, Syt IV signal was not elevated in plaque periphery (20 μm wide tissue ring around Syt IV-enriched corona) or in interplaque tissue (Figure 2E). At the protein level, Syt IV up-regulation was confirmed also with Western immunoblotting analysis, which showed ~ 1.5 fold increased Syt IV levels in the cortex and hippocampus (1.1 ± 0.1 a.u.) compared to control mice (0.7 ± 0.04 a.u.) (Figure 2E-F, unpaired two-tailed t-test, *P* = 0.011).

### Syt IV is present in dystrophic neurons around the β-amyloid plaques

**Figure 3:**
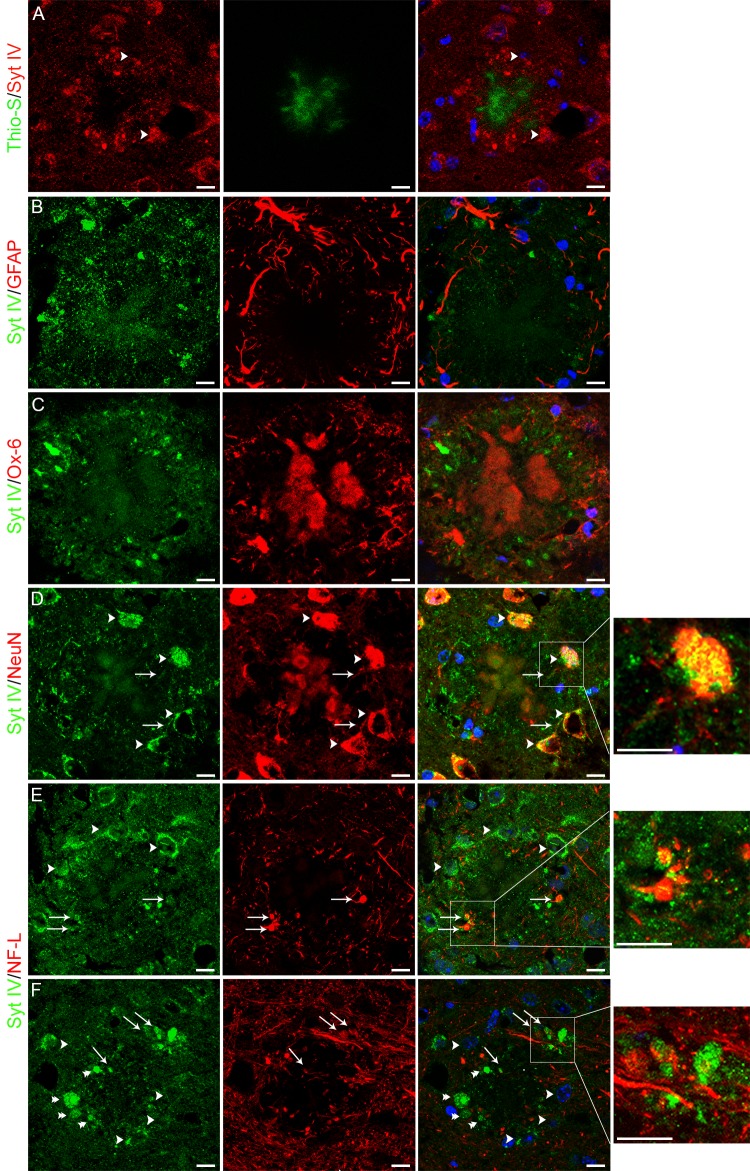
Up-regulation of Synaptotagmin IV (Syt IV) around the β-amyloid plaques occurs in dystrophic neurons. Representative images of Syt IV immunostaining counterstained with Thioflavin S (**A**) and double immunofluorescence labeling of Syt IV-GFAP (**B**), SytIV-Ox-6 (**C**), Syt IV-NeuN (**D**), and Syt IV-neurofilaments (NF-L, **E-F**) in the cortex of transgenic mice. Nuclei (blue) were counterstained with DAPI. Boxed areas in images D-F are shown in magnified view. Up-regulated Syt IVwas localized on β-amyloid plaque periphery forming a corona composed of irregularly shaped to spherical structures (**A**) and Syt IV immunofluorescent signal enclosing cell nuclei indicating Syt IV up-regulation in neuronal soma (**A**, **D-F**, arrowheads). β-amyloid plaques were surrounded by GFAP-immunoreactive astrocytes (**B**), Ox-6-immunoreactive microglial cells (**C**), and NeuN-positive neuronal cells (**D**). Syt IV-positive were only neuronal cells (**D**). Up-regulated Syt IV co-localized with amyloid plaque associated NeuN-positive neuronal soma (arrowheads, **D**) and processes (arrows, **D**). Spherical Syt IV-positive structures co-localized with bulb-like neurofilaments-positive dystrophic neurites (arrows, **E-F**), however some Syt IV-immunoreactive dystrophic neurites were not neurofilaments-positive (double arrowheads, **F**). Scale bars, 10 μm.

Immunofluorescence labeling also showed Syt IV up-regulation around core amyloid plaques in transgenic cortex (Figure 3). Counterstaining of Syt IV immunolabeled transgenic mice brain sections with Thioflavin S demonstrated that elevated Syt IV was surrounding β-amyloid fibrils (Figure 3A) in a form of irregularly shaped and spherical structures or was surrounding cell nuclei, indicating that amyloid-associated neuronal soma abundantly express Syt IV (Figure 3A, 3D-F). Up-regulated Syt IV did not overlap with Thioflavin S labeled β-amyloid deposits. GFAP-positive astrocytes (Figure 3B) and Ox-6-positive microglial cell (Figure 3C) were enclosing/surrounding β-amyloid plaques; however on most micrographs no astrocyte or microglial cell was positive for Syt IV. Up-regulated Syt IV signal was visible in the NeuN labeled neuronal soma and processes (Figure 3D) and also in dystrophic neurites surrounding amyloid plaques. Several Syt IV-positive spherical structures around plaques co-localized with neurofilament-positive bulb-like structures indicating dystrophic neurites (Figure 3E). Amyloid plaques were also surrounded by bulb-like Syt IV-immunoreactive dystrophic neurites that were not neurofilaments-positive (Figure 3F).

**Figure 4:**
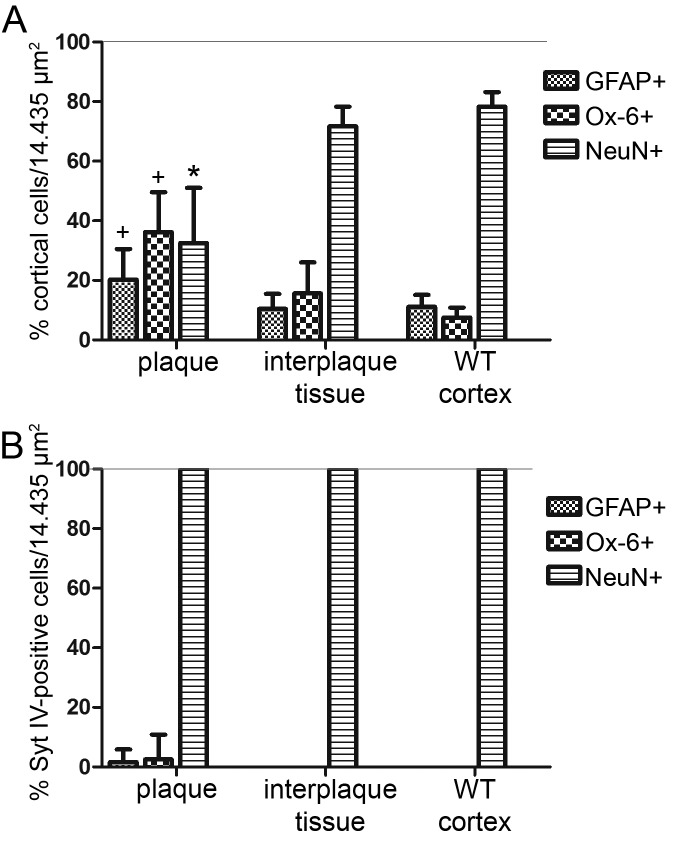
Quantitative analysis of double immunofluorescence staining in the cortex of Tg2576 and wild type (WT) mice. (**A**) The percentage of glial fibrillary acid protein (GFAP), Ox-6, or NeuN-positive cells in plaque, interplaque regions, and WT cortex expressed as percentage of all cortical cells. The number of GFAP-positive and Ox-6-positive cells around amyloid plaques was significantly higher than in interplaque and WT cortical tissue. On the contrary, the number of NeuN-positive cells decreased around plaques (two-way ANOVA followed by Tukey multiple comparison test, ^+^significantly higher than in interplaque tissue and WT cortex, *P* < 0.05; *significantly lower than in interplaque tissue and WT cortex; *P* < 0.001, (**A**). (**B**) The percentage of GFAP, Ox-6, or NeuN-positive cells stained with Syt IV antibody in plaque, interplaque regions, and WT cortex. The vast majority of GFAP- and Ox-6-positive cells surrounding amyloid plaques were not positive for up-regulated Syt IV. There were no astrocytes or microglial cells positive for Syt IV in interplaque tissue or in WT cortex. All NeuN-positive cells were Syt IV-positive in all cortices. (**B**). Bars represent the standard deviation of the mean; Tg2576 mice (n = 4) and WT mice (n = 4).

Quantitative analysis of double fluorescence staining showed an increased number of GFAP-positive cells around amyloid plaques as compared with interplaque areas (two-way ANOVA, Tukey post hoc test, *P* = 0.021, Figure 4A) and WT mouse cortex (two-way ANOVA, Tukey post hoc test, *P* = 0.018, Figure 4A). Similarly the number of Ox-6-positive cells was higher around plaques than in interplaque areas (two-way ANOVA, Tukey post hoc test, *P* < 0.001, Figure 4A) and WT mouse cortex (two-way ANOVA, Tukey post hoc test, *P* < 0.001, Figure 4A). However, the majority of amlyoid plaques were surrounded by GFAP-positive and Ox-6-positive cells that were not positive for Syt IV; only 1.6 ± 4.3% (mean±standard deviation) of GFAP-positive cells were Syt IV-positive and 2.5 ± 6.4% (mean±standard deviation) of Ox-6-positive cells were Syt IV-positive (Figure 4B). We did not find astrocytes or microglial cells positive for Syt IV in interplaque regions or WT cortex (Figure 4B).

In contrast to the increased number of glial cells around plaques, the number of NeuN-positive cells significantly decreased around plaques as compared with interplaque cortical areas in Tg2576 mice (two-way ANOVA, Tukey post hoc test, *P* < 0.001, Figure 4A) and WT cortical areas (two-way ANOVA, Tukey post hoc test, *P* < 0.001, Figure 4A). All NeuN-positive cells around plaques were positive for Syt IV. Similarly all NeuN-positive cells in interplaque tissue and in non-transgene mouse cortex were always Syt IV-positive (Figure 4B).

## Discussion

We showed that in the WT mice brain cortex Syt IV was expressed exclusively in neurons, which is in agreement with one of the previous reports (28). Similarly, Zhang et al (29) found Syt IV to be expressed in neurons and not in other cell types in the hypothalamus. Mittelsteadt et al (28) found only a minority of astrocytes expressing Syt IV. Nevertheless, Syt IV protein was found in astrocytes by *in vitro* experiments under non-pathological conditions (10,26,27). This discrepancy between the results of *in vitro* experiments and this study could be a consequence of our inability to detect Syt IV in non-activated astrocytes in brain tissue of WT mice using the double immunofluorescence method, and of a higher background typical for tissue staining. Furthermore, our results show that in WT mice cortical neurons Syt IV is localized in cell body and along axons and dendrites. This is consistent with the findings of Ibata et al (33), who reported that Syt IV was present in the Golgi apparatus and along both axons and dendrites in the mouse brain. Similar Syt IV subcellular distribution in neurons has also been demonstrated *in vitro* (11,34,35).

The result of immunohistochemical staining is in accordance with our previous publication showing that up-regulation of Syt IV in aged Tg2576 mice was found only in the proximity of β-amyloid plaques (14). There several pathological features of AD develop: dystrophic neurites, reactive astrocytes, and activated microglial cells (32,36-38). The present study demonstrated that only around 2% of glial cells surrounding plaques were showing Syt IV-immunoreactivity, which is why Syt IV was probably not involved in the processes of the β-amyloid plaque-associated activation of astrocytes or microglial cells. On the contrary, double immunofluorescence staining revealed that Syt IV protein was up-regulated in neurons; neuronal soma, and dystrophic neurites. The greatest part of Syt IV-immunoreactive signal was in the form of spherical structures, in neurofilaments-positive bulb-like dystrophic neurites. Such structures were found by many other authors using different types of staining (immunolabeling of neurofilament triplet proteins, α-sinuclein, tau, green fluorescent protein (GFP) labeled neurons) as dystrophic neurites – axonal and dendritic swellings mainly found in the proximity of the plaques in Tg2576 mice (4,39-41). Neurofilaments-positive dystrophic neurites represent only a subgroup of dystrophic neurites. These are composed of different neurochemical constituents and can be immunolabeled with different markers that accumulate within dystrophic neurites (neurofilaments, amyloid precursor protein, paired helical filament tau, some synaptic markers etc) (42-45). Hence, we presume that Syt IV-positive spherical structures that did not co-localize with neurofilaments were present in other dystrophic neurite compartments.

To clarify whether up-regulated Syt IV protein in Tg2576 mice is the cause or a consequence of neuron degeneration, we compared the intensity of Syt IV immunohistochemistry signal obtained from the WT cortex, cortex with plaques, and cortex of Tg2576 mice between the plaques (resembling the pre-symptomatic conditions). The Syt IV signal was up-regulated only around amyloid plaques indicating that its up-regulation is probably a consequence of neurodegeneration. Neurodegeneration in our study was shown by a decreased number of neurons around plaques as compared to interplaque and WT cortices.

Amyloid plaques have toxic effect on the surrounding neurons in AD mice (3,46). β-amyloid neuropathology resembles the biochemical and morphological changes that follow physical axonal injury (47). Identical reactive changes that subsequently lead to an attempt at regenerative sprouting by damaged axons were observed in experimental models of structural axonal injury and neuritic pathology associated with amyloid plaques. Considering that Syt IV expression is increased during neuronal injury (16,19-21,23,25) and that Syt IV has been recognized to be involved in synaptic plasticity (12,13,48-50), Syt IV up-regulation could be a part of aberrant regenerative response occurring in amyloid-associated neurons (47). Another feature of plaque-induced neurodegeneration is the accumulation of synaptic proteins, which was proposed to result from synaptic vesicle accumulation within dystrophic neurites (45). Several synaptic markers and other proteins were found to accumulate in dystrophic neurites: Syt I, synaptophysin, SV2, pentaxin 1, prion protein (45,51,52). Morfini et al (8) found that accumulation of organelles in dystrophic neurites reflected a disruption of axonal transport. Fast axonal transport of membrane bound organelles including those containing APP, synaptophysin, syntaxin, and others (53,54) was reported to be inhibited in various AD mouse models. Diminished degradation and axonal transport leads to the accumulation of organelles in autophagic vacuoles within large swellings along dystrophic and degenerating neurites (6). As Syt IV is normally present in axons and as we demonstrated a strong up-regulation of Syt IV immunosignal in dystrophic neurites this up-regulation could also be due to the accumulation of Syt IV protein as a consequence of the neuronal metabolic disturbance.

Previous studies revealed that Syt IV mRNA expression could be up-regulated in several animal models of neurodegeneration – for Parkinson’s disease, brain ischemia, Huntington’s disease, and epilepsy (14-25). Basically, Syt IV mRNA up-regulation in these models could be ascribed to two different mechanisms. The first mechanism is an excessive stimulation of receptors and the second is neurodegeneration. The first was reflected in the stimulation of D1 dopamine receptors in hypersensitive striatum of parkinsonian rats and the stimulation of glutamate receptors in kainate animal model of epilepsy (24,25). According to the second mechanism, Syt IV up-regulation induced by neuronal degeneration is probably taking place after brain ischemia, in Huntington’s disease model and, as shown by this study, in Tg2576 mice model for AD (14). To determine the extent and the mechanisms of the contribution of Syt IV to the pathology characteristic of AD a more detailed study on Tg2576 mice is needed such as the use of young, presymptomatic animals or the use of primary neuronal cultures exposed to the amyloid. Nevertheless, our study for the first time directly shows up-regulation of Syt IV in neurons in any animal model of neurodegeneration. We believe that elucidating the detailed mechanism of Syt IV induction caused by β-amyloid plaques may be important for understanding the neuronal pathology relevant for cognitive impairment in AD.
